# Association between serum chlamydial antibody levels and tubal infertility in tertiary health facility in South-East Nigeria: a case-control study

**DOI:** 10.4314/gmj.v55i3.2

**Published:** 2021-09

**Authors:** Augustine D Onyeabochukwu, Emmanuel O Izuka, Onyema A Onyegbule, Chiemeka C Onumajuru, Uchenna T Ejelonu, Chukwunonyerem P Duke-Onyeabo, Chinelo E Obiora-Izuka, Uchenna I Nwagha

**Affiliations:** 1 Department of Obstetrics and Gynaecology, Federal Medical Centre, Owerri, Nigeria; 2 Department of Obstetrics and Gynaecology, University of Nigeria Teaching Hospital, Ituku-Ozalla, Enugu. Nigeria; 3 Department of Paediatrics, Enugu State University Teaching Hospital-Parklane, Enugu, Nigeria; 4 Department of Paediatrics, University of Nigeria Teaching Hospital, Ituku-Ozalla, Enugu, Nigeria

**Keywords:** Tubal factor infertility, chlamydial infections, chlamydial IgG antibodies

## Abstract

**Objectives:**

This study evaluates the association between genital Chlamydial infection and tubal factor infertility in a tertiary health facility in South-East Nigeria.

**Design:**

This was a case-control analytical study.

**Setting:**

Gynaecology Clinic and Maternity Unit of the Department of Obstetrics and Gynaecology of the Federal Medical Centre (FMC), Owerri, Imo State, Nigeria.

**Participants:**

Ninety-six (96) women with confirmed tubal factor infertility served as the cases, and 96 women with normal intra-uterine pregnancy matched in age served as the control.

**Data Collection/Intervention:**

A structured questionnaire was used to extract information on the sociodemographic data and the sexual history of the participants. About 2mls of blood was collected, the blood was allowed to clot, and the sera were used for the test.

**Statistical analysis/Main outcome measure:**

Pearson Chi-square, Fisher's exact test, likelihood ratio and multivariate logistic regression were used to determine risk associations and identify factors independently related to tubal factor infertility. P-value < 0.05 was considered significant.

**Results:**

The sociodemographic characteristics of both cases and control did not differ (P = 0.975). The Chlamydial antibody seropositivity was significantly higher in the cases than the control 78(81.2%) versus 13(13.5%) respectively {(P < 0.001; OR (95% CI) = 27.7(12.7–60.2)}. Only lower abdominal pain {(P = 0.011); OR (95% CI) = 4.3(1.4–13.3)}; was independently associated with tubal factor infertility.

**Conclusion:**

Tubal factor infertility is strongly associated with chlamydial IgG antibodies, and a history of lower abdominal pain significantly predicted tubal factor infertility.

**Funding:**

The authors paid the cost of procuring the anti-chlamydial ELIZA test kits, plain sample bottles, syringes, gloves and other consumables and stationaries

## Introduction

Despite the high fertility rates and “high rates of pregnancy wastages” in sub-Saharan Africa, infertility was recently described as rampant. It constituted a severe social problem in this part of the world.^1^ This may not be unconnected because a high premium is placed on childbearing, and any childless woman is seen as not being a real woman on the African continent.

Apart from the financial drain that a couple looking for a child might suffer, the woman is frequently stigmatized, resulting in neglect, social isolation, domestic violence and ultimately divorce or polygamy.^2^–^4^

In Sub-Saharan Africa, infertility is the commonest reason for a consultation in a Gynaecological clinic, and tubal factor is the commonest cause of infertility in this sub-region.^2^,^5^–^6^

*Chlamydia trachomatis* infection is the most common bacterial sexually transmitted infection.^7^–^8^ Tubal disease from *Chlamydia trachomatis* infection occurs because of repeated infections of the upper genital tract, usually subclinical^6^,^9^ and asymptomatic.

These repeated infections often culminate in the destruction of the cilia within the tubes, which eventually leads to bilateral tubal blockage of varying degrees^9^ leading to long term consequences such as pelvic inflammatory disease (PID), tubal damage and infertility, ectopic pregnancy and chronic pelvic pain.^7^,^10^ Studies have shown that Chlamydial infection causes as much as 50% of salpingitis and tubal factor infertility.^7^The infection caused by Chlamydia trachomatis usually results in the formation of antibodies that are detectable in the serum of affected patients even after the infection has resolved.^11^

Using tissue culture for the diagnosis of Chlamydial infection is usually expensive and time consuming^9^,^12^, and in most cases, unaffordable in resource-limited settings. Therefore, using the serological detection of current or previous chlamydial genital infections to predict a possible tubal blockage will save time and money.

Several studies have shown a correlation between chlamydial immunoglobulin G (IgG) and tubal factor infertility.^11^,^13^–^15^ All the studies employed ELIZA for testing the chlamydia antibody, HSG and laparoscopy were used to check tubal patency. However, all these were Asian and European based studies. Although tubal factor infertility is the commonest cause of female infertility,^6^,^7^,^9^ minimal data exist on its association with prior *Chlamydia trachomatis* infection in our subregion. This study aims at evaluating the association between chlamydia immunoglobulin G antibodies and patients with tubal factor infertility.

## Methods

### Study sites

The study was conducted at the Gynaecological Clinic and Maternity Unit of the Department of Obstetrics and Gynaecology of the Federal Medical Centre (FMC), Owerri, South-East Nigeria. FMC Owerri is a tertiary health care facility undertaking postgraduate medical training. Located in Owerri Metropolis, the capital of Imo State of Nigeria, it has about 500,000 people, with an estimated 140,000 (28%) of these women of reproductive age.^16^ Owerri is an urban city with up to 5 higher institutions (universities, polytechnics and monotechnics) in and around it. It has mainly civil servants and businessmen and women as her inhabitants.

### Study population

The study comprised 192 subjects of two equal groups. Group One, which is the case, consisted of 96 patients with confirmed tubal factor infertility using hysterosalpingography. Group two is the control made up of 96 women with intrauterine pregnancy who conceived through natural means, thus generally believed they had no tubal factor infertility. Using the hysterosalpingogram, tubal damage was defined as unilateral or bilateral dye obstruction or hydrosalpinx as reported by a radiologist. Laparoscopy and dye test were not done for these patients because of their unavailability in our centre at the time of this study.

### Eligibility criteria

Women with a history of inability to conceive at least one year duration who have evidence of tubal damage demonstrated by hysterosalpingography while women with normal pregnancy who were matched in age with the cases were selected as controls.

### Exclusion criteria

Women with tubal factor infertility due to previous abdominopelvic surgery and pregnant women that conceived via in-vitro fertilization and embryo transfer.

### Recruitment and sampling

After informed consent was obtained, a structured questionnaire was given to each participant, which they completed individually and returned immediately. The information gathered using the questionnaire included personal and socio-demographic data such as age, ethnicity, religion, occupation, educational status, marital status, type of marriage, and the number of previous pregnancies. The sexual history such as age at coitarche, number of lifetime sexual partners and history of sexually transmitted diseases and pelvic inflammatory disease were explored. For instance, patients were asked if there was a past history of abnormal vaginal discharge or past history of lower abdominal pain, which made them seek medical attention.

### Study design

This was a case-control study involving 96 patients attending the Gynaecology clinic with confirmed tubal factor infertility who satisfied the inclusion criteria. They were recruited consecutively until the required sample size was reached. Once a case of tubal infertility was identified, the next eligible consenting pregnant woman in the antenatal clinic who conceived through natural means and who was of similar age was selected as a control. We assumed that once a woman conceives spontaneously without assistance that the tubes are patent. Therefore, no tubal patency test was done for those women that were used as control.

A structured questionnaire was used to extract information on the sociodemographic data and the sexual history of the participants.

After obtaining informed consent, about 2mls of blood was collected from each patient, by venepuncture of a peripheral vein, into a universal specimen bottle. The blood was allowed to clot, and the sera were used for the test. The test was carried out on the day of collection of the sample, but where the sera were not used on the day of collection; it was stored in the freezer compartment at - 20°C until it is ready to be analyzed. The sample was tested for chlamydia immunoglobulin G antibodies using Abcam's anti-Chlamydia trachomatis IgG Human invitro Enzyme-Linked Immunosorbent Assay (ELISA) kit (ab108720), which is a rapid diagnostic kit designed for the accurate qualitative measurement of IgG class antibodies against *Chlamydia trachomatis* in human serum or plasma. The Abcam (ab108720) ELISA (the USA, 2013) kit has a sensitivity of >95% and specificity of 91.3% for detecting Chlamydia trachomatis IgG antibodies in human serum or plasma.

The sample size was determined using the prevalence of a study done in Port-Harcourt on serum immunoglobulin G antibody to Chlamydia trachomatis in sub-fertile women where 74% case of the group versus 51% of the control group were positive for chlamydia antibody (CAT).^17^ The minimum sample size for this study was calculated using an equation by Whitley E and Ball J. 18 A minimum of 87 cases in each group was required at a 95% confidence interval and a 5% margin of error to obtain 80% power for the study. Taking this into cognizance and adjusting for a 10% non-response rate, 96 cases were recruited for each group.

### Ethical clearance

Permission for this study was obtained from the Health Ethics and Research Committee of FMC, Owerri, with reference number FMC/OW/HREC/35. Voluntariness in participation in the research and confidentiality was ensured throughout the study. Written informed consent was obtained from each participant before recruitment.

### Data Analysis

Statistical analysis was done using Statistical Package for Social Sciences (SPSS) version 23 software (Chicago IL USA). Descriptive statistics (means and standard deviation) were determined for continuous variables, while frequencies and percentages were used for categorical variables. Pearson Chi-square and Fisher's exact tests, which are non-parametric inferential statistics were used to determine the risk association between variables and tubal factor infertility.

A stepwise backward multivariate logistic regression was used to identify factors that were independently related to tubal factor infertility. A P-value less than 0.05 was considered significant.

## Results

Table 1 shows that the ages of the subjects and controls were matched with most of the study participants within the age bracket of 30 and 39 years (*χ*^2^ = 0.215, p = 0.975). The mean ages were 34.07 ± 3.96 for subjects and 34.08 ± 4.01 for controls.

**Table 1:** Socio-demographic characteristics of the study population

	Cases n (%)	Controls n (%)	*χ* ^2^	P value
***Age group***				
**25 – 29**	11 (11.5)	13 (13.5)	0.215	0.975
**30 – 34**	42 (43.8)	40 (41.7)		
**35 – 39**	30 (31.3)	30 (31.3)		
**≥40**	13 (13.5)	13 (13.5)		
***Marital status***				
**Married**	96 (100.0)	96 (100.0)	NA	NA
***Type of marriage***				
**Monogamous**	91 (94.8)	95 (99.0)	2.753	0.097
**Polygamous**	5 (5.2)	1 (1.0)		
***Level of Education***				
**Primary**	1 (1.0)	0 (0.0)	8.931	0.011
**Secondary**	22 (22.9)	8 (8.3)		
**Tertiary**	73 (76.0)	88 (91.7)		
***Occupation***				
**Housewife**	3 (3.1)	5 (5.2)	5.471	0.242
**Student**	12 (12.5)	14 (14.6)		
**Trader**	42 (43.8)	30 (31.3)		
**Civil servant**	34 (35.4)	45 (46.9)		
**Others**	5 (5.2)	2 (2.1)		
***Religion***				
**Christianity**	96 (100.0)	96 (100.0)	NA	NA
***Tribe***				
**Igbo**	96 (100.0)	96 (100.0)	NA	NA

Table 2 shows a greater number of pregnancies, number of children, number of spontaneous abortions, age of Coitarche, number of the sexual partner and abnormal vaginal discharge among pregnant controls (p < 0.05). The prevalence of chlamydial antibodies among the subject was 81.3%, while the prevalence was 13.5% among the controls. Chlamydial antibodies were significantly associated with tubal factor infertility (p < 0.001, OR = 27.7, 95% C.I = 12.7 – 60.2). Women with tubal factor infertility were much more likely to have chlamydial antibodies than the pregnant controls.

**Table 2:** Sexual and reproductive behaviour of the study population

	Cases n (%)	Controls n (%)	*χ* ^2^	P value
***No. of pregnancies***				
**≤2**	57 (59.4)	29 (30.2)	16.513	<0.001
**>2**	39 (40.6)	67 (69.8)		
***No. of children***				
**0**	85 (88.5)	3 (3.1)	141.063	<0.001
≥1	11 (11.5)	93 (96.9)		
***No. of spontaneous abortions***				
**0**	86 (89.6)	72 (75.0)	7.005	0.008
**≥1**	10 (10.4)	24 (25.0)		
***No. of induced abortions***				
**0**	4 (4.2)	74 (77.1)	105.803	<0.001
**≥1**	92 (95.8)	22 (22.9)		
***Age of Coitarche***				
**<20**	51 (53.1)	7 (7.3)	47.827	<0.001
**≥20**	45 (46.9)	89 (92.7)		
***No. of sexual partner***				
**1 – 2**	7 (7.3)	45 (46.9)	38.084	<0.001
**≥3**	89 (92.7)	51 (53.1)		
***Abnormal vaginal discharge***				
**Yes**	93 (96.9)	96 (100.0)	3.048	0.081
**No**	3 (3.1)	0 (0.0)		
***Lower abdominal pain***				
**Yes**	67 (69.8)	16 (16.7)	55.200	<0.001
**No**	29 (30.2)	80 (83.3)		
***Dyspareunia***				
**Yes**	2 (2.1)	0 (0.0)	2.021	0.155
**No**	94 (97.9)	96 (100.0)		
***Dysmenorrhea***				
**Yes**	8 (8.3)	1 (1.0)	5.712	0.017
**No**	88 (91.7)	95 (99.0)		
***Source of treatment (hospital/clinic)***				
**Yes**	82 (85.4)	85 (88.5)	0.414	0.520
**No**	14 (14.6)	11 (11.5)		

All study participants were married, Christians, Igbos and predominantly Monogamous. More controls attained a tertiary level of education than the subjects (*χ*^2^ = 8.931, p = 0.011). Trading and civil service were the predominant occupation in both groups.

Table 3 shows that the number of sexual partners and lower abdominal pain have a significant association with chlamydial antibodies among patients with tubal factor infertility (P < 0.05). Patients with 1 to 2 sexual partners were less likely to have chlamydial antibodies than those with three or more sexual partners (p = 0.016, OR = 0.1, 95% C.I = 0.0 – 0.7). Patients with lower abdominal pain were more likely to have chlamydial antibodies than those without lower abdominal pain (p = 0.003, OR = 5.2, 95% C.I = 1.8 – 15.5).

**Table 3:** Association between sexual/reproductive behaviour and chlamydial antibodies among patients with tubal factor infertility

	Chlamydial Antibody				
	Positive n (%)	Negative n (%)	P value	OR	95% C.I for OR
***No. of pregnancies***					
**≤2**	43 (55.1)	14 (77.8)	0.087	0.4	0.1 – 1.2
**>2**	35 (44.9)	4 (22.2)			
***No. of children***					
**0**	70 (89.7)	15 (83.3)	0.446	1.8	0.4 – 7.4
**≥1**	8 (10.3)	3 (16.7)			
***No. of spontaneous abortions***					
**0**	69 (88.5)	17 (94.4)	0.464	0.5	0.1 – 3.9
**≥1**	9 (11.5)	1 (5.6)			
***No. of induced abortions***					
**0**	2 (2.6)	2 (11.1)	0.133	0.2	0.0 – 1.7
**≥1**	76 (97.4)	16 (88.9)			
***Age of Coitarche***					
**<20**	45 (57.7)	6 (33.3)	0.068	2.8	1.0 – 8.0
**≥20**	33 (42.3)	12 (66.7)			
***No. of sexual partner***					
**1 – 2**	3 (3.8)	4 (22.2)	0.016	0.1	0.0 – 0.7
**≥3**	75 (96.2)	14 (77.8)			
***Abnormal vaginal discharge***					
**Yes**	76 (97.4)	17 (94.4)	0.521	2.2	0.2 – 26.1
**No**	2 (2.6)	1 (5.6)			
***Lower abdominal pain***					
**Yes**	60 (76.9)	7 (38.9)	0.003	5.238	1.8 – 15.5
**No**	18 (23.1)	11 (61.1)			
***Dyspareunia***					
**Yes**	0 (0.0)	2 (11.1)	NA	NA	NA
**No**	78 (10.0)	16 (88.9)			
***Dysmenorrhea***					
**Yes**	5 (6.4)	3 (16.7)	0.171	0.3	0.1 – 1.6
**No**	73 (93.6)	15 (83.3)			
***Source of treatment (hospital/clinic)***					
**Yes**	67 (85.9)	15 (83.3)	0.781	1.2	0.3 – 5.0
**No**	11 (14.1)	3 (16.7)			

NA = Not applicable

Only lower abdominal pain was a significant predictor of chlamydial antibodies after including all valid variables in a Backward Stepwise multivariate logistic regression model (p = 0.011, OR = 4.3, 95% C.I = 1.4 – 13.3).

## Discussion

This study showed that 81.2% of those with tubal factor infertility and only 13.5% of the pregnant controls were positive for chlamydial immunoglobin G antibodies. This finding agrees with the report of other studies which showed that women with tubal factor infertility are more likely to have chlamydial immunoglobin G antibodies when compared with pregnant women without tubal blockage,^10^,^19^,^20^,^21^ emphasizing the role of past genital *Chlamydia trachomatis* infection on tubal damage. Notable among these studies are Olaleye O et al. in Lagos, Nigeria, and Malik A, et al. in India.

In their research, Olaleye O et al. found that 65.7% of those with tubal infertility were positive while only 10.8% of those without tubal damage were positive for chlamydial IgG antibody.^21^ Also, Malik A et al. found that 72.7% of women with tubal infertility had positive antichlamydial IgG antibodies while only 6.7% of the pregnant controls were positive for antichlamydial antibodies. These suggest the heavy impact genital C. trachomatis infection has on the pathogenesis of tubal pathology and infertility in developing countries in which Nigeria and India belong to.^20^

Our study found that women with tubal factor infertility are much more likely to have Chlamydial antibodies than the pregnant controls. This finding is comparable to those of previous studies.^17^, ^21^, ^22^, ^23^ The findings point to the possible role of CAT in infertility as a screening tool for assessing tubal damage with or without the result of hysterosalpingography (HSG). This is even more so in our environment where laparoscopy, which is the gold standard for assessing tubal damage, is still not widely in use as it is limited by its non-availability and cost. Because Chlamydial antibody testing is relatively cheap and noninvasive when compared to HSG or laparoscopy, it might be helpful, especially in low resource settings like ours, as a screening tool in infertility workup as some authors have recommended doing HSG for those with negative or low CAT titres and laparoscopy for those with high CAT titres.^17^, ^24^ This is because studies have shown that chlamydial IgG antibodies are quantitatively related to the severity of tubal damage.^17^, ^24^ Although we did not do a quantitative analysis of chlamydial antibody testing in our study, we found that majority of the cases had bilateral cornual tubal obstruction on HSG, and they were the majority who tested positive for chlamydial IgG antibody.

The bivariate analysis results showed that women with tubal factor infertility were more likely to be nulliparous than the pregnant controls. This finding is in keeping with Siemer J et al., which linked nulliparity to tubal damage and infertility.^25^ Nulliparity may be a consequence of tubal injury rather than a cause since those with tubal impairment may not conceive through natural means. Also, this study showed that women with tubal factor infertility had their first sexual intercourse at a significantly younger age than the pregnant controls. This finding agrees with other studies which associated early coitarche with Chlamydia trachomatis infection and subsequent tubal damage.^2^, ^25^

The reason for this association is not apparent. However, it is believed that early coitarche predisposes the adolescent girl to contract sexually transmitted diseases, including *Chlamydia trachomatis*. The adolescent girl is also less likely to use a barrier method of contraception during sex and is more likely to conceal any symptom of sexually transmitted infections resulting in persistent subclinical infection with its attendant sequelae. This suggests the need to educate adolescents and young women on the need to practice safer sex and also seek prompt medical care if they notice any abnormal symptom in their system.

We found that, abnormal vaginal discharge, and dysmenorrhea, which could be symptoms of pelvic *Chlamydia trachomatis* infection, were not significantly different in both the cases and the controls, which contrasts to some other studies.^24^, ^26^, ^27^ The reason for this is not apparent. Still, it is possible that most of the pelvic infections that caused the tubal damage in this setting were subclinical but were severe enough to cause tubal damage. It is also possible that the patients may not have remembered past events in detail, which is one of the disadvantages of using a questionnaire interview, as was the case in this study.

When the factors which had some correlation with Chlamydial infection and tubal infertility on bivariate analysis were subjected to multivariate logistic regression, the result was that the effects of a higher number of sexual partners were attenuated. However, only lower abdominal pain was still associated with tubal infertility. This shows the importance of lower abdominal pain as it concerns genital Chlamydial infection and tubal factor infertility. This study also showed that genital *Chlamydia trachomatis* infection might not be the only cause of tubal damage in tubal factor infertility, as demonstrated by 18.8% of the women with tubal factor infertility who were seronegative for anti-chlamydial antibody. These might be due to Neisseria gonorrhoeae, Mycoplasma species and endometriosis.^28^, ^29^ Therefore, further research may be required to examine the role of other sexually transmitted organisms in the pathogenesis of tubal injury in our environment.

Using questionnaire interview and self-reporting of confidential events like sexual and reproductive behaviors may be limited by recall bias and the deliberate withholding of information. However, this was mitigated by a strong reassurance of utmost confidentiality before data collection. The reliance on the accuracy of hysterosalpingography alone in identifying tubal blockage is another limitation as some false-positive results may have occurred if compared with laparoscopy and dye test. Again, the laboratory analysis relied on serology in making a diagnosis of past chlamydial infection. Serology is less sensitive than nucleic acid amplification tests (NAAT), which is now considered the gold standard in diagnosing chlamydial diseases. Unfortunately, NAAT assays are rarely used in our environment because of cost, availability, and technical complexity. However, the Abcam ELISA kit used in this study has a sensitivity of greater than 95% and specificity of greater than 91% when compared with NAAT. The current study is hospital-based research and cannot be generalized however, it forms a pivot for future community-based research.

## Conclusion

In conclusion, women with tubal factor infertility had a significant association with past *Chlamydia trachomatis* infection. Anti-chlamydial immunoglobulin G antibodies, which is evidence of previous Chlamydia trachomatis infection, was associated with more than twenty-eightfold increase odds of tubal factor infertility. Also, lower abdominal pain has an independent association with tubal infertility.
